# Acridinium 6-carb­oxy­pyridine-2-carboxyl­ate monohydrate

**DOI:** 10.1107/S1600536810053791

**Published:** 2011-01-15

**Authors:** Zohreh Derikvand, Marilyn M. Olmstead, Jafar Attar Gharamaleki

**Affiliations:** a Department of Chemistry, Faculty of Sciences, Islamic Azad University, Khorramabad Branch, Khorramabad, Iran; b Department of Chemistry, University of California, One Shields Avenue, Davis, CA 95616, USA; c Young Researchers Club, Islamic Azad University, North Tehran Branch, Tehran, Iran

## Abstract

The title compound, C_13_H_10_N^+^·C_7_H_4_NO_4_^−^·H_2_O or (acrH)^+^(pydcH)^−^·H_2_O, is a monohydrate of acridinium cations and a mono-deprotonated pyridine-2,6-dicarb­oxy­lic acid. The structure contains a range of non-covalent inter­actions, such as O—H⋯O, O—H⋯N and N—H⋯O hydrogen bonds, as well as π–π stacking [range of centroid–centroid distances = 3.4783 (5)–3.8059 (5) Å]. The N—H⋯O hydrogen bond between the donor acridinium cation and the carboxyl­ate acceptor is particularly strong. The average separation between the π-stacked acridinium planes is 3.42 (3) Å.

## Related literature

For structures of acridinium salts, see: Aghabozorg *et al.* (2010 ▶); Attar Gharamaleki *et al.* (2010 ▶); Derikvand *et al.* (2009 ▶, 2010 ▶); Shaameri *et al.* (2001 ▶); Tabatabaee *et al.* (2009 ▶).
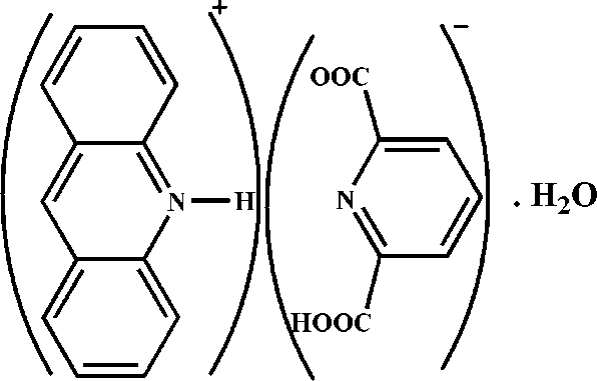


## Experimental

### 

#### Crystal data

C_13_H_10_N^+^·C_7_H_4_NO_4_^−^·H_2_O
*M* *_r_* = 364.35
Triclinic, 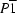

*a* = 7.4842 (3) Å
*b* = 8.6850 (3) Å
*c* = 13.0305 (4) Å
α = 100.266 (3)°
β = 93.851 (2)°
γ = 97.766 (2)°
*V* = 822.16 (5) Å^3^
*Z* = 2
Mo *K*α radiation
μ = 0.11 mm^−1^
*T* = 90 K
0.32 × 0.23 × 0.17 mm

#### Data collection

Bruker SMART APEXII diffractometer
Absorption correction: multi-scan (*SADABS*; Sheldrick, 1996 ▶) *T*_min_ = 0.966, *T*_max_ = 0.982
11632 measured reflections
4403 independent reflections
4034 reflections with *I* > 2σ(*I*)
*R*_int_ = 0.011

#### Refinement

*R*[*F* ^2^ > 2σ(*F* ^2^)] = 0.035
*wR*(*F* ^2^) = 0.105
*S* = 1.07
4403 reflections
308 parameters
All H-atom parameters refined
Δρ_max_ = 0.48 e Å^−3^
Δρ_min_ = −0.20 e Å^−3^

### 

Data collection: *APEX2* (Bruker, 2009 ▶); cell refinement: *SAINT* (Bruker, 2009 ▶); data reduction: *SAINT*; program(s) used to solve structure: *SHELXS97* (Sheldrick, 2008 ▶); program(s) used to refine structure: *SHELXL97* (Sheldrick, 2008 ▶); molecular graphics: *XP* in *SHELXTL* (Sheldrick, 2008 ▶); software used to prepare material for publication: *SHELXL97*.

## Supplementary Material

Crystal structure: contains datablocks I, global. DOI: 10.1107/S1600536810053791/hg2777sup1.cif

Structure factors: contains datablocks I. DOI: 10.1107/S1600536810053791/hg2777Isup2.hkl

Additional supplementary materials:  crystallographic information; 3D view; checkCIF report

## Figures and Tables

**Table 1:** Hydrogen-bond geometry (Å, °)

*D*—H⋯*A*	*D*—H	H⋯*A*	*D*⋯*A*	*D*—H⋯*A*
O4—H4*A*⋯O5	0.869 (17)	1.958 (17)	2.7604 (10)	152.9 (16)
O4—H4*A*⋯N2	0.869 (17)	2.182 (17)	2.6646 (10)	114.6 (14)
O5—H5*A*⋯O1	0.849 (18)	2.016 (18)	2.8421 (10)	164.0 (16)
O5—H5*B*⋯O2^i^	0.845 (18)	2.134 (18)	2.9255 (10)	155.8 (16)
N1—H1⋯O2	1.031 (17)	1.555 (18)	2.5859 (9)	178.6 (16)

Symmetry code: (i) 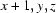.

**Fig. 1.**
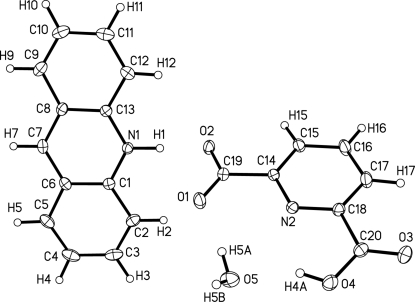
Molecular structure of (acrH)+(pydcH)-. H2O. Displacement ellipsoids are drawn at 50% probability level.

**Fig. 2.**
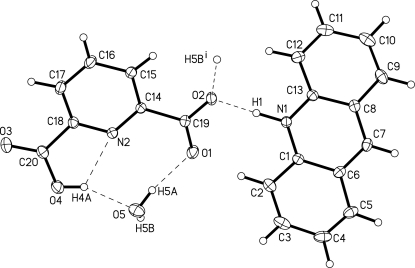
Hydrogen bonding interactions. Symmetry code: i = x - 1, y, z.

**Fig. 3.**
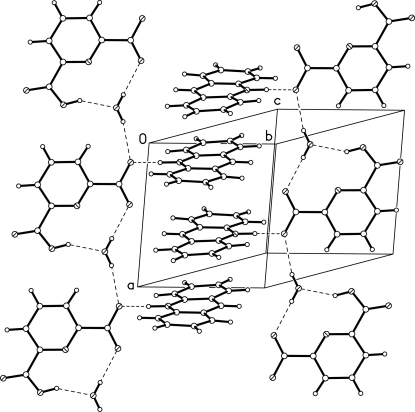
π–π Stacking interactions between cationic fragments and their link to anionic chains as viewed along [0 1 1].

## supplementary crystallographic information

### Comment

We have reported a number of crystal structures of protonated acridine and pyridine dicarboxylates (Derikvand *et al.*, 2009, 2010; Aghabozorg *et al.*, 2010; Attar Gharamaleki *et al.*, 2010; Tabatabaee *et al.*, 2009). Many other examples of acridinium salts are known, and they have π–π stacking of the acridinium ions and various types of hydrogen bonding in common. The molecular structure of the title compound, the 1:1 salt of acridinium and pydridine-2,6-dicarboylate is illustrated in Fig. 1. The crystal structure shows one of the protons of the two carboxylic groups has been transferred to the nitrogen atom of the acridine molecule.

As expected, bond lengths of the –CO_2_ groups reflect the presence or lack of an acidic H atom. At distances of 1.2403 (11) Å and 1.2806 (1)) Å, respectively, the O1—C19 and O2—C19 bond lengths are much closer to equality than O3—C20 and O4—C20, at 1.226 (11) Å and 1.3305 (11) Å. However, we can also point out that the O2—C19 bond is slightly longer than the O1—C19 bond, possibly due to there being two classical hydrogen bonds involving O2 and only one involving O1 (see Table 1). In fact, one of the hydrogen bonds for O2 can be classified as a very strong hydrogen bond, with an N···O distance of 2.5859 (9) Å. In a similar structure involving the acridinium salt of isophthalate (Shaameri *et al.*, 2001), the analogous arrangement of cation and anion gives rise to a similar short hydrogen bond with N···O distance of 2.553 (2) Å. A depiction of the hydrogen bonded motif involving anion and cation fragments and water molecules is presented in Fig. 2. The hydrogen bonds between the water molecule and O2 serve to link the anions into a chain along the *a* axis direction. Symmetry code: i = *x* - 1, *y*, *z*.

Additional noncovalent interactions cause the structure to form a self assembled system. In the structure π–π stacking interactions between the acridinium ions average 3.42[3]Å (average deviation in square brackets). Sideways strong hydrogen bonds between O2 and the the proton of acridine gather the π-stack and the anionic chain together as shown in Fig. 3.

### Experimental

A solution of pyridine-2,6-dicarboxylic acid (167 mg, 1 mmol) in water (10 ml) was added to a solution of acridine(179 mg, 1 mmol) in methanol (5 ml) and stirring for 30 minutes, a clear solution was obtained (Scheme 1). Yellow-gold block crystals suitable for X-ray crystallography were produced by slow evaporation of the solvent at room temperature after a week.

### Refinement

All hydrogen atoms were freely refined.

### Crystal data

**Table:**

C_13_H_10_N^+^·C_7_H_4_NO_4_^−^·H_2_O	*Z* = 2
*M**_r_* = 364.35	*F*(000) = 380
Triclinic, *P*1	*D*_x_ = 1.472 Mg m^−^^3^
Hall symbol: -P 1	Mo *K*α radiation, λ = 0.71073 Å
*a* = 7.4842 (3) Å	Cell parameters from 7626 reflections
*b* = 8.6850 (3) Å	θ = 2.6–32.9°
*c* = 13.0305 (4) Å	µ = 0.11 mm^−^^1^
α = 100.266 (3)°	*T* = 90 K
β = 93.851 (2)°	Block, yellow
γ = 97.766 (2)°	0.32 × 0.23 × 0.17 mm
*V* = 822.16 (5) Å^3^	

### Data collection

**Table:**

Bruker SMART APEXII diffractometer	4403 independent reflections
Radiation source: fine-focus sealed tube	4034 reflections with *I* > 2σ(*I*)
graphite	*R*_int_ = 0.011
Detector resolution: 8.3 pixels mm^-1^	θ_max_ = 29.1°, θ_min_ = 2.6°
ω scans	*h* = −10→10
Absorption correction: multi-scan (*SADABS*; Sheldrick, 1996)	*k* = −11→11
*T*_min_ = 0.966, *T*_max_ = 0.982	*l* = −17→17
11632 measured reflections	

### Refinement

**Table:**

Refinement on *F*^2^	Primary atom site location: structure-invariant direct methods
Least-squares matrix: full	Secondary atom site location: difference Fourier map
*R*[*F*^2^ > 2σ(*F*^2^)] = 0.035	Hydrogen site location: inferred from neighbouring sites
*wR*(*F*^2^) = 0.105	All H-atom parameters refined
*S* = 1.07	*w* = 1/[σ^2^(*F*_o_^2^) + (0.0621*P*)^2^ + 0.1884*P*] where *P* = (*F*_o_^2^ + 2*F*_c_^2^)/3
4403 reflections	(Δ/σ)_max_ < 0.001
308 parameters	Δρ_max_ = 0.48 e Å^−^^3^
0 restraints	Δρ_min_ = −0.20 e Å^−^^3^

### Special details

**Table:**

Geometry. All e.s.d.'s (except the e.s.d. in the dihedral angle between two l.s. planes) are estimated using the full covariance matrix. The cell e.s.d.'s are taken into account individually in the estimation of e.s.d.'s in distances, angles and torsion angles; correlations between e.s.d.'s in cell parameters are only used when they are defined by crystal symmetry. An approximate (isotropic) treatment of cell e.s.d.'s is used for estimating e.s.d.'s involving l.s. planes.
Refinement. Refinement of *F*^2^ against ALL reflections. The weighted *R*-factor *wR* and goodness of fit *S* are based on *F*^2^, conventional *R*-factors *R* are based on *F*, with *F* set to zero for negative *F*^2^. The threshold expression of *F*^2^ > σ(*F*^2^) is used only for calculating *R*-factors(gt) *etc*. and is not relevant to the choice of reflections for refinement. *R*-factors based on *F*^2^ are statistically about twice as large as those based on *F*, and *R*- factors based on ALL data will be even larger.

### Fractional atomic coordinates and isotropic or equivalent isotropic displacement parameters (Å^2^)

**Table:**

	*x*	*y*	*z*	*U*_iso_*/*U*_eq_	
O1	0.49045 (9)	0.63220 (8)	0.25575 (5)	0.02109 (15)	
O2	0.19185 (9)	0.63670 (8)	0.23357 (5)	0.01783 (14)	
O3	0.62172 (10)	0.08319 (8)	−0.08226 (5)	0.02271 (16)	
O4	0.75469 (9)	0.23901 (8)	0.06409 (6)	0.02201 (15)	
H4A	0.735 (2)	0.320 (2)	0.1097 (13)	0.043 (4)*	
O5	0.81245 (10)	0.49602 (9)	0.22739 (6)	0.02321 (16)	
H5A	0.721 (2)	0.544 (2)	0.2252 (13)	0.044 (4)*	
H5B	0.907 (2)	0.560 (2)	0.2244 (13)	0.043 (4)*	
N1	0.22761 (10)	0.86086 (8)	0.39668 (6)	0.01351 (15)	
H1	0.215 (2)	0.771 (2)	0.3318 (14)	0.049 (5)*	
N2	0.46280 (10)	0.38054 (8)	0.09346 (5)	0.01356 (15)	
C1	0.29254 (11)	0.83725 (10)	0.49119 (6)	0.01346 (16)	
C2	0.33756 (12)	0.68689 (11)	0.50193 (7)	0.01783 (18)	
H2	0.3209 (19)	0.6010 (17)	0.4401 (11)	0.029 (3)*	
C3	0.40456 (13)	0.66579 (12)	0.59791 (8)	0.02161 (19)	
H3	0.432 (2)	0.5632 (18)	0.6068 (11)	0.034 (4)*	
C4	0.43460 (13)	0.79225 (13)	0.68579 (8)	0.0226 (2)	
H4	0.486 (2)	0.7719 (18)	0.7514 (12)	0.034 (4)*	
C5	0.39282 (12)	0.93759 (12)	0.67742 (7)	0.01935 (18)	
H5	0.418 (2)	1.0241 (17)	0.7378 (11)	0.031 (4)*	
C6	0.31603 (11)	0.96397 (10)	0.57969 (6)	0.01448 (16)	
C7	0.26325 (11)	1.10795 (10)	0.56689 (7)	0.01559 (17)	
H7	0.2804 (19)	1.1941 (17)	0.6252 (11)	0.028 (3)*	
C8	0.19056 (11)	1.12820 (10)	0.46937 (7)	0.01433 (17)	
C9	0.13152 (12)	1.27198 (11)	0.45221 (8)	0.01948 (18)	
H9	0.139 (2)	1.3590 (17)	0.5148 (11)	0.031 (3)*	
C10	0.06669 (13)	1.28604 (12)	0.35436 (9)	0.0227 (2)	
H10	0.026 (2)	1.3839 (18)	0.3435 (12)	0.037 (4)*	
C11	0.05946 (13)	1.15875 (12)	0.26824 (8)	0.02219 (19)	
H11	0.016 (2)	1.1712 (18)	0.1989 (12)	0.037 (4)*	
C12	0.11412 (12)	1.01882 (11)	0.28081 (7)	0.01838 (18)	
H12	0.1125 (19)	0.9325 (17)	0.2221 (11)	0.028 (3)*	
C13	0.17760 (11)	1.00027 (10)	0.38252 (6)	0.01340 (16)	
C14	0.31558 (11)	0.44843 (9)	0.11517 (6)	0.01310 (16)	
C15	0.14892 (12)	0.39585 (11)	0.05742 (7)	0.01748 (17)	
H15	0.0454 (18)	0.4460 (16)	0.0776 (10)	0.025 (3)*	
C16	0.13303 (13)	0.26913 (12)	−0.02620 (7)	0.0224 (2)	
H16	0.015 (2)	0.2304 (19)	−0.0658 (12)	0.039 (4)*	
C17	0.28452 (13)	0.19891 (11)	−0.04975 (7)	0.02011 (19)	
H17	0.280 (2)	0.1079 (17)	−0.1077 (11)	0.032 (4)*	
C18	0.44547 (11)	0.25928 (10)	0.01274 (6)	0.01459 (16)	
C19	0.33787 (12)	0.58364 (10)	0.20896 (6)	0.01463 (16)	
C20	0.61355 (12)	0.18562 (10)	−0.00691 (7)	0.01689 (17)	

### Atomic displacement parameters (Å^2^)

**Table:**

	*U*^11^	*U*^22^	*U*^33^	*U*^12^	*U*^13^	*U*^23^
O1	0.0156 (3)	0.0221 (3)	0.0213 (3)	0.0021 (2)	−0.0019 (2)	−0.0053 (2)
O2	0.0154 (3)	0.0174 (3)	0.0187 (3)	0.0044 (2)	0.0012 (2)	−0.0031 (2)
O3	0.0247 (3)	0.0232 (3)	0.0206 (3)	0.0092 (3)	0.0066 (3)	−0.0007 (3)
O4	0.0159 (3)	0.0220 (3)	0.0267 (4)	0.0062 (2)	0.0003 (3)	−0.0013 (3)
O5	0.0150 (3)	0.0245 (4)	0.0307 (4)	0.0020 (3)	0.0008 (3)	0.0078 (3)
N1	0.0133 (3)	0.0131 (3)	0.0137 (3)	0.0024 (2)	0.0012 (2)	0.0011 (2)
N2	0.0139 (3)	0.0140 (3)	0.0130 (3)	0.0031 (2)	0.0014 (2)	0.0025 (2)
C1	0.0110 (3)	0.0146 (4)	0.0150 (4)	0.0015 (3)	0.0024 (3)	0.0034 (3)
C2	0.0157 (4)	0.0162 (4)	0.0233 (4)	0.0040 (3)	0.0048 (3)	0.0060 (3)
C3	0.0154 (4)	0.0251 (5)	0.0297 (5)	0.0067 (3)	0.0061 (3)	0.0153 (4)
C4	0.0149 (4)	0.0363 (5)	0.0199 (4)	0.0036 (4)	0.0020 (3)	0.0145 (4)
C5	0.0147 (4)	0.0290 (5)	0.0139 (4)	0.0005 (3)	0.0011 (3)	0.0052 (3)
C6	0.0117 (4)	0.0182 (4)	0.0127 (4)	−0.0002 (3)	0.0014 (3)	0.0025 (3)
C7	0.0145 (4)	0.0149 (4)	0.0153 (4)	−0.0005 (3)	0.0025 (3)	−0.0012 (3)
C8	0.0124 (4)	0.0128 (4)	0.0173 (4)	0.0006 (3)	0.0028 (3)	0.0020 (3)
C9	0.0169 (4)	0.0134 (4)	0.0286 (5)	0.0026 (3)	0.0049 (3)	0.0040 (3)
C10	0.0172 (4)	0.0194 (4)	0.0352 (5)	0.0051 (3)	0.0037 (4)	0.0126 (4)
C11	0.0169 (4)	0.0288 (5)	0.0237 (4)	0.0034 (3)	0.0001 (3)	0.0132 (4)
C12	0.0164 (4)	0.0230 (4)	0.0158 (4)	0.0024 (3)	−0.0001 (3)	0.0045 (3)
C13	0.0112 (3)	0.0141 (4)	0.0149 (4)	0.0015 (3)	0.0017 (3)	0.0029 (3)
C14	0.0145 (4)	0.0134 (4)	0.0114 (3)	0.0028 (3)	0.0012 (3)	0.0020 (3)
C15	0.0147 (4)	0.0221 (4)	0.0145 (4)	0.0056 (3)	−0.0003 (3)	−0.0011 (3)
C16	0.0162 (4)	0.0297 (5)	0.0172 (4)	0.0049 (3)	−0.0026 (3)	−0.0062 (3)
C17	0.0188 (4)	0.0231 (4)	0.0156 (4)	0.0041 (3)	0.0012 (3)	−0.0042 (3)
C18	0.0156 (4)	0.0152 (4)	0.0135 (4)	0.0041 (3)	0.0031 (3)	0.0022 (3)
C19	0.0159 (4)	0.0135 (4)	0.0139 (4)	0.0023 (3)	0.0011 (3)	0.0009 (3)
C20	0.0161 (4)	0.0170 (4)	0.0185 (4)	0.0040 (3)	0.0041 (3)	0.0038 (3)

### Geometric parameters (Å, °)

**Table:**

O1—C19	1.2403 (11)	C6—C7	1.3952 (12)
O2—C19	1.2806 (10)	C7—C8	1.3980 (12)
O3—C20	1.2126 (11)	C7—H7	0.955 (14)
O4—C20	1.3305 (11)	C8—C13	1.4256 (11)
O4—H4A	0.869 (17)	C8—C9	1.4282 (12)
O5—H5A	0.849 (18)	C9—C10	1.3655 (14)
O5—H5B	0.845 (18)	C9—H9	1.002 (14)
N1—C1	1.3533 (11)	C10—C11	1.4204 (15)
N1—C13	1.3538 (11)	C10—H10	0.972 (16)
N1—H1	1.031 (17)	C11—C12	1.3665 (13)
N2—C18	1.3350 (11)	C11—H11	0.970 (16)
N2—C14	1.3415 (11)	C12—C13	1.4215 (12)
C1—C2	1.4199 (12)	C12—H12	0.969 (14)
C1—C6	1.4283 (11)	C14—C15	1.3885 (12)
C2—C3	1.3680 (13)	C14—C19	1.5194 (11)
C2—H2	0.984 (14)	C15—C16	1.3901 (12)
C3—C4	1.4208 (15)	C15—H15	0.968 (14)
C3—H3	0.966 (15)	C16—C17	1.3850 (13)
C4—C5	1.3617 (14)	C16—H16	0.977 (16)
C4—H4	0.969 (15)	C17—C18	1.3908 (12)
C5—C6	1.4303 (12)	C17—H17	0.987 (15)
C5—H5	0.975 (14)	C18—C20	1.5032 (12)
			
C20—O4—H4A	112.1 (11)	C9—C10—C11	120.43 (9)
H5A—O5—H5B	109.3 (16)	C9—C10—H10	119.8 (9)
C1—N1—C13	122.44 (7)	C11—C10—H10	119.7 (9)
C1—N1—H1	120.2 (10)	C12—C11—C10	121.37 (9)
C13—N1—H1	117.3 (10)	C12—C11—H11	119.0 (9)
C18—N2—C14	117.75 (7)	C10—C11—H11	119.6 (9)
N1—C1—C2	119.91 (8)	C11—C12—C13	119.08 (9)
N1—C1—C6	119.81 (8)	C11—C12—H12	121.8 (8)
C2—C1—C6	120.28 (8)	C13—C12—H12	119.1 (8)
C3—C2—C1	119.03 (9)	N1—C13—C12	119.76 (8)
C3—C2—H2	121.8 (8)	N1—C13—C8	119.91 (7)
C1—C2—H2	119.2 (8)	C12—C13—C8	120.32 (8)
C2—C3—C4	121.31 (9)	N2—C14—C15	122.24 (8)
C2—C3—H3	119.9 (9)	N2—C14—C19	116.69 (7)
C4—C3—H3	118.8 (9)	C15—C14—C19	121.05 (7)
C5—C4—C3	120.73 (8)	C14—C15—C16	119.29 (8)
C5—C4—H4	121.3 (9)	C14—C15—H15	119.3 (8)
C3—C4—H4	118.0 (9)	C16—C15—H15	121.4 (8)
C4—C5—C6	120.05 (9)	C17—C16—C15	118.93 (8)
C4—C5—H5	119.8 (9)	C17—C16—H16	121.7 (9)
C6—C5—H5	120.1 (9)	C15—C16—H16	119.3 (9)
C7—C6—C1	118.53 (8)	C16—C17—C18	117.69 (8)
C7—C6—C5	122.96 (8)	C16—C17—H17	122.0 (9)
C1—C6—C5	118.52 (8)	C18—C17—H17	120.3 (9)
C6—C7—C8	120.73 (8)	N2—C18—C17	124.10 (8)
C6—C7—H7	119.2 (9)	N2—C18—C20	115.58 (7)
C8—C7—H7	120.0 (9)	C17—C18—C20	120.31 (8)
C7—C8—C13	118.48 (8)	O1—C19—O2	125.44 (8)
C7—C8—C9	123.10 (8)	O1—C19—C14	119.23 (8)
C13—C8—C9	118.42 (8)	O2—C19—C14	115.33 (7)
C10—C9—C8	120.32 (9)	O3—C20—O4	121.35 (8)
C10—C9—H9	122.8 (8)	O3—C20—C18	122.73 (8)
C8—C9—H9	116.9 (8)	O4—C20—C18	115.92 (7)
			
C13—N1—C1—C2	178.05 (7)	C11—C12—C13—N1	178.23 (8)
C13—N1—C1—C6	−2.15 (12)	C11—C12—C13—C8	−2.42 (13)
N1—C1—C2—C3	179.41 (8)	C7—C8—C13—N1	2.91 (12)
C6—C1—C2—C3	−0.39 (13)	C9—C8—C13—N1	−178.14 (7)
C1—C2—C3—C4	−1.84 (13)	C7—C8—C13—C12	−176.43 (8)
C2—C3—C4—C5	1.74 (14)	C9—C8—C13—C12	2.51 (12)
C3—C4—C5—C6	0.66 (14)	C18—N2—C14—C15	0.51 (12)
N1—C1—C6—C7	2.80 (12)	C18—N2—C14—C19	178.61 (7)
C2—C1—C6—C7	−177.40 (8)	N2—C14—C15—C16	−0.32 (14)
N1—C1—C6—C5	−177.12 (7)	C19—C14—C15—C16	−178.35 (8)
C2—C1—C6—C5	2.68 (12)	C14—C15—C16—C17	−0.15 (15)
C4—C5—C6—C7	177.28 (8)	C15—C16—C17—C18	0.39 (15)
C4—C5—C6—C1	−2.80 (13)	C14—N2—C18—C17	−0.24 (13)
C1—C6—C7—C8	−0.59 (12)	C14—N2—C18—C20	−178.89 (7)
C5—C6—C7—C8	179.32 (8)	C16—C17—C18—N2	−0.21 (15)
C6—C7—C8—C13	−2.20 (12)	C16—C17—C18—C20	178.38 (9)
C6—C7—C8—C9	178.91 (8)	N2—C14—C19—O1	5.15 (12)
C7—C8—C9—C10	178.03 (8)	C15—C14—C19—O1	−176.72 (8)
C13—C8—C9—C10	−0.87 (13)	N2—C14—C19—O2	−173.96 (7)
C8—C9—C10—C11	−0.86 (14)	C15—C14—C19—O2	4.17 (12)
C9—C10—C11—C12	0.97 (14)	N2—C18—C20—O3	−173.96 (8)
C10—C11—C12—C13	0.68 (14)	C17—C18—C20—O3	7.33 (14)
C1—N1—C13—C12	178.61 (7)	N2—C18—C20—O4	6.37 (11)
C1—N1—C13—C8	−0.74 (12)	C17—C18—C20—O4	−172.34 (8)

### Hydrogen-bond geometry (Å, °)

**Table:**

*D*—H···*A*	*D*—H	H···*A*	*D*···*A*	*D*—H···*A*
O4—H4A···O5	0.869 (17)	1.958 (17)	2.7604 (10)	152.9 (16)
O4—H4A···N2	0.869 (17)	2.182 (17)	2.6646 (10)	114.6 (14)
O5—H5A···O1	0.849 (18)	2.016 (18)	2.8421 (10)	164.0 (16)
O5—H5B···O2^i^	0.845 (18)	2.134 (18)	2.9255 (10)	155.8 (16)
N1—H1···O2	1.031 (17)	1.555 (18)	2.5859 (9)	178.6 (16)

Symmetry codes: (i) *x*+1, *y*, *z*.
